# The Readiness Potential Correlates with Action-Linked Modulation of Visual Accuracy

**DOI:** 10.1523/ENEURO.0085-22.2022

**Published:** 2022-11-22

**Authors:** Alessandro Benedetto, Hao Tam Ho, Maria Concetta Morrone

**Affiliations:** 1 Department of Translational Research on New Technologies in Medicine and Surgery, University of Pisa, 56123 Pisa, Italy; 2 Department of Brain and Cognitive Sciences and Center for Visual Science, University of Rochester, New York 14627, NY; 3 Department of Neuroscience, Psychology, Pharmacology and Child Health, University of Florence, Florence 50153, Italy

**Keywords:** action and perception, EEG, efference copy, ERP, motor-induced visual modulation, readiness potential

## Abstract

Visual accuracy is consistently shown to be modulated around the time of the action execution. The neural underpinning of this motor-induced modulation of visual perception is still unclear. Here, we investigate with EEG whether it is related to the readiness potential, an event-related potential (ERP) linked to motor preparation. Across 18 human participants, the magnitude of visual modulation following a voluntary button press was found to correlate with the readiness potential amplitude measured during visual discrimination. Participants’ amplitude of the readiness potential in a purely motor-task was also found to correlate with the extent of the motor-induced modulation of visual perception in the visuomotor task. These results provide strong evidence that perceptual changes close to action execution are associated with motor preparation processes and that this mechanism is independent of task contingencies. Further, our findings suggest that the readiness potential provides a fingerprint of individual visuomotor interaction.

## Significance Statement

Vision and motor action need to be closely synchronized. Performing, or even programming, an action changes our visual perception: the ability to discriminate visual stimuli typically decreases transiently at the time of execution of a movement such as a button press. In the present study we demonstrate that the magnitude of this perceptual modulation is predicted by the strength of an EEG premotor signal that precedes the action execution, the readiness potential: the greater the magnitude of this signal, the weaker the visual modulation. We suggest that this mechanism may be exploited by the brain to synchronize and coordinate action and perception over time.

## Introduction

To act effectively on the sensory information that we receive every moment of our daily life, action and perception require precise temporal coordination. The neural mechanisms underlying sensorimotor interactions are complex and not fully understood. However, they likely involve a premotor signal (Helmholtz, 1866; Sperry, 1950; von Holst and Mittelstaedt, 1950), that prepares the visual system for the sensory consequences of a self-initiated action. This may account for a range of phenomena, including saccadic suppression, where visual sensitivity is suppressed for a short time around saccadic execution (50 ms before to 50 ms after; Burr et al., 1994). The function of this suppression may be to maintain visual stability during eye movements by suppressing transient and spurious motion signals caused by the rotation of the eyeball. However, similar suppressive effects have been reported for a variety of sensorimotor tasks involving different body parts (Bays et al., 2005; Cardoso-Leite et al., 2010; Weiss et al., 2011; Stenner et al., 2014a), suggesting that modulation of perception around the time of an action is a fundamental and general characteristic of visuomotor interactions.

Findings from functional MRI scans show that visually evoked responses in the primary visual cortex are reduced for stimuli immediately following a voluntary button press (Straube et al., 2017; Benedetto et al., 2021). Importantly, this modulation begins during motor preparation, well before action onset (Rolfs et al., 2013; Gutteling et al., 2015; Tomassini et al., 2017; Gallivan et al., 2019; Monaco et al., 2020). This latter finding is consistent with results from transcranial magnetic stimulation, showing that stimulating the supplementary and presupplementary motor areas involved in the preparation and planning of voluntary movements induce sensorimotor attenuation (Haggard and Whitford, 2004; Voss et al., 2006). Interestingly, these areas and primary motor cortex contribute to the generation of an event-related potential (ERP), called the readiness potential (Kornhuber and Deecke, 1965; Vaughan et al., 1968), which emerges 1–2 s before action execution and is closely related to motor preparation and planning (Libet et al., 1983). This slow negative-going wave, which is also present before saccadic eye movements (Barlow and Cigánek, 1969), consists of two subcomponents: (1) an early bilateral component that starts in the presupplementary and supplementary motor area and appears shortly after in the lateral premotor cortices and (2) a late component that arises around 500–400 ms before action onset, contralateral to the site of the movement, possibly in the primary motor cortex (Neshige et al., 1988; Ikeda et al., 1992; Shibasaki and Hallett, 2006). Although the late component is considered motor-specific, an early study by McAdam and Rubin (1971) found that modulations of its amplitude (∼300–100 ms before action execution) were associated with differences in confidence of the performance for stimuli presented immediately after a voluntary action: on average high confidence was associated with higher readiness potential. This early study, while pointing to an interesting link between action and sensory processing, was limited in several aspects. First, the stimuli were consistently presented at a fixed delay from action onset, possibly contaminating the readiness potential response with sensory prediction signals. More importantly, the task was a simple localization task, and no association was found between individuals’ performance and readiness potential amplitude. The involvement of the readiness potential in modulating sensory process has been demonstrated in more recent studies on the anticipation of sensory consequences following self-initiated actions (Reznik et al., 2018; Wen et al., 2018; Travers et al., 2021). Readiness potential has also been linked to intentional binding (Jo et al., 2014), as well as in temporal recalibration of motor-sensory signals (Cai et al., 2018). For instance, the readiness potential amplitude correlates with the perceived asynchrony between the action onset and its perceptual consequence (Jo et al., 2014), an effect known as intentional binding (Haggard et al., 2002). Interestingly, the readiness potential has been proposed as an indirect measure of the efference copy signals (Reznik et al., 2018; Vercillo et al., 2018; Wen et al., 2018; Travers et al., 2021), which may mediate the modulation of visual accuracy around the time of action execution.

The present study investigates the link between the readiness potential and differences in visual accuracy at around the time of action execution and demonstrates that the amplitude of the readiness potential is associated with the modulation of visual perceptual accuracy of stimuli presented around the onset of the action.

## Materials and Methods

### Participants

A total of 18 volunteers (including two authors; mean age ± SD: 27 ± 2, 10 women and 8 men) participated in the study. The sample size was chosen based on previous experiments in the same field of research (Stenner et al., 2014a; Tomassini et al., 2017). The experimental procedures are in line with the Declaration of Helsinki and were approved by the local regional ethics committee. Written informed consent was obtained from all participants. This includes consent to process and preserve the data, and publish them anonymously.

### Apparatus

The visual stimuli were generated with Psychtoolbox for MATLAB (MATLAB r2017b, The MathWorks, Inc.) and displayed on a γ-calibrated Display++ monitor (Cambridge Research System, resolution of 1920 × 1080 pixels, refresh rate of 120 Hz). A custom response box was connected with a Ni-DAQ USB-6001 to the experimental computer to record button-press timing and send triggers to the EEG device.

EEG was recorded using a 32 active-channel wireless g.Nautilus system, with a sampling rate of 500 Hz. The scalp electrodes were positioned according to the 10–20 international system and the reference electrode on the right earlobe. The impedance was checked before each recording and kept below 50 kΩ.

### Stimulus and procedure

The experiment consisted of two tasks: a visuomotor, and motor-only task, completed in separate blocks over two recording sessions on different days. In session 1, participants performed the motor-only task (100 trials) and two blocks of the visuomotor task (162 trials per block). In session 2, they performed another two blocks of the visuomotor task (162 trials per block). Before each session, participants completed a training block to familiarize themselves with the tasks. In total, we collected 648 trials per participant for the visuomotor task and 100 for the motor-only task. Two (out of 18) participants who participated in the initial pilot phase of the experiment underwent three additional sessions (six blocks), bringing the total number of visuomotor trials to 1620 each. Given the result congruency between the initial two sessions with the later three sessions, we pooled all trials for these two participants.

#### Visuomotor task

**Figure 1.**
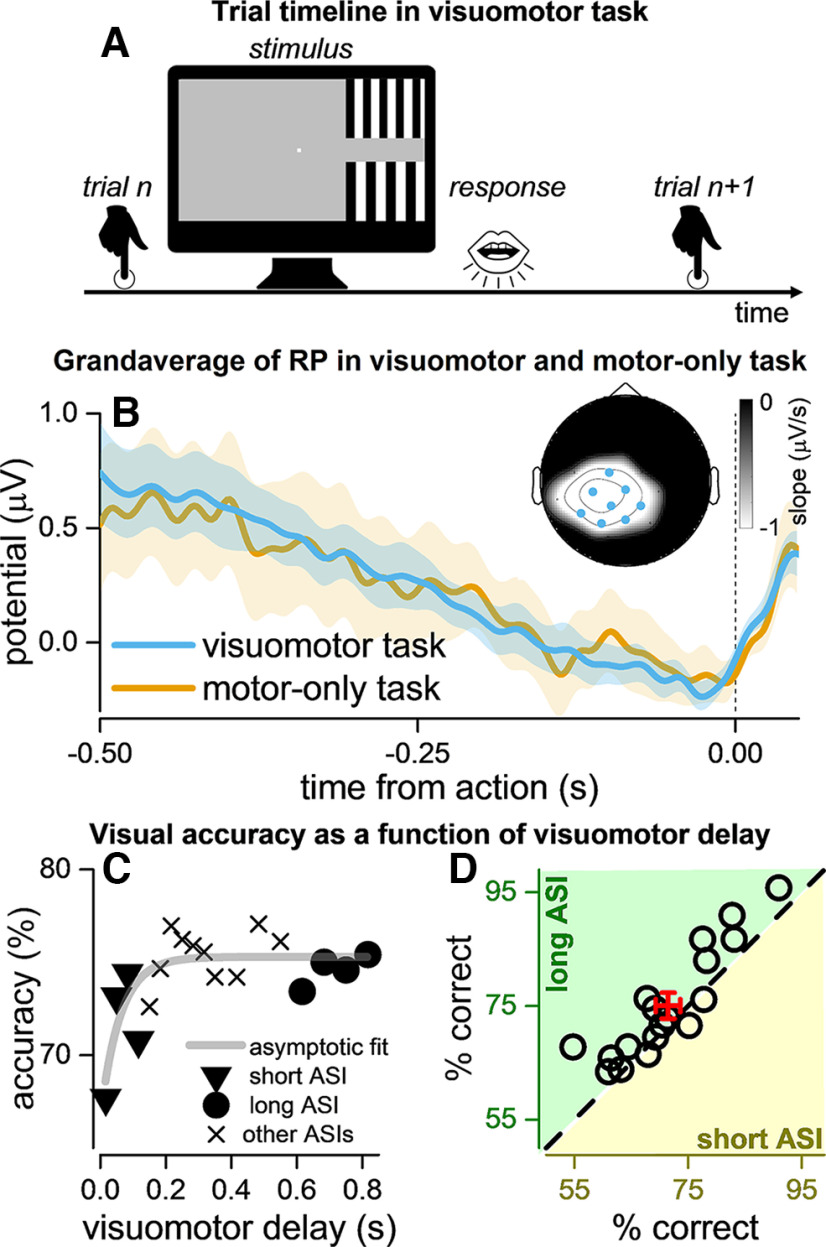
Experimental paradigm, readiness potential activity, and temporal dynamic of visual accuracy. ***A***, Schematic timeline of an example trial in the visuomotor task. Participants pressed a key to start the trial. The visual stimulus comprised two gratings with different spatial frequency, displayed after a random delay on the left or right hemifield. Participants had to indicate which grating (upper or lower) had the higher spatial frequency by means of a verbal response. ***B***, Time course of readiness potential relative to the button press. The light blue and orange lines show the grand-averaged ERPs in the visuomotor and motor-only tasks, respectively, relative to action onset (0 s). Colored shaded areas indicate the standard error. The ERPs reflect the average activity at eight electrodes of interest: FC1, C3, CZ, CP5, CP1, CP2, P3, highlighted in blue in the inset. The intensity map of the topographical EEG plot shows the ERP slope in the interval −0.5 to −0.02 s from the keypress for all electrodes. ***C***, Temporal dynamic of visual accuracy as a function of visuomotor delays, for the aggregate observer (*n* = 18). Triangles and circles mark accuracies for short and long ASIs, respectively. Gray thick line shows the best asymptotic exponential fit to the data. ***D***, Perceptual accuracy for stimuli presented after short ASIs (delays < 120 ms, *x*-axis in dark yellow) and long ASIs (delays > 600 ms, *y*-axis in green). The open circles represent the individual accuracies (*n* = 18); the black dashed line is the equality line. The red cross shows the group mean accuracy ± 1 SEM. Modulation of visual perception was estimated as the difference between the individual long and short ASIs accuracy. See Extended Data Figure 1-1 for readiness potential activity when using an earlier baseline (−0.5 to −0.4 s), Extended Data Figures 1-2 and 1-3 for eye movements analyses, and Extended Data Figure 1-4 for the topography of the slope of the ERPs in the motor-only condition.

The visual stimulus in the visuomotor condition comprised two vertical gratings (32° × 16°, 50% contrast, random phase) presented for 8.3 ms (one frame) randomly in the right or left visual field. The two gratings were always presented in the same hemifield and placed 3° left/right from a small fixation square displayed at the center of the screen. The gratings had a fixed spatial frequency of 1 and 1.1 c/° (10% difference), randomly presented in the upper or lower part of the monitor (see Fig. 1*A*). Participants were instructed to maintain their fixation on the small center square and pressed a key (with the index finger of the right hand) to start each trial. The visual stimulus was presented with 18 possible stimulus delays after the button press, chosen randomly on each trial in the interval between 16 and 816 ms to avoid a stereotypical allocation of subject attention to very late or very early after the action onset. The random stimulus presentation to the left or right visual field and the short stimulus exposure aimed to minimize the number of saccades coinciding with the stimulus which could impair discrimination performance. The delays had a denser sampling in the first 350 ms from button press (33-ms bins) and sparser sampling at later delays (66-ms bins). The task was to indicate which grating (upper or lower) had the higher spatial frequency. Participants were instructed to maintain fixation throughout the trial, and to wait at least 1.5 s from the stimulus onset before giving a verbal response, coded by the experimenter. They waited at least another 1.5 s before starting the next trial. Participants were trained to adhere to the trial timing. The mean interval (±1 SD) between successive button presses was 5.46 ± 0.88 s. Participants waited on average 2.6 ± 1 s from stimulus onset before providing a verbal response. They were warned when their responses occurred too early, but the trials were not excluded from the analysis (<1% of trials had responses below 1 s).

10.1523/ENEURO.0085-22.2022.f1-1 Extended Data Figure 1-1 Readiness potential activity with baseline computed between −0.5 and 0.4 s. ***A***, Time course of readiness potential relative to the button press. The light blue and orange lines show the grand-averaged ERPs in the visuomotor and motor-only tasks, respectively, relative to action onset (0 s). Colored shaded areas indicate the standard error. The ERPs reflect the average activity at eight electrodes of interest: FC1, C3, CZ, CP5, CP1, CP2, P3, highlighted in blue in the inset. The intensity map of the topographical EEG plot shows the ERP slope in the interval −0.5 and −0.02 s from the keypress, for all electrodes. Download Figure 1-1, TIF file.

**Figure 2.**
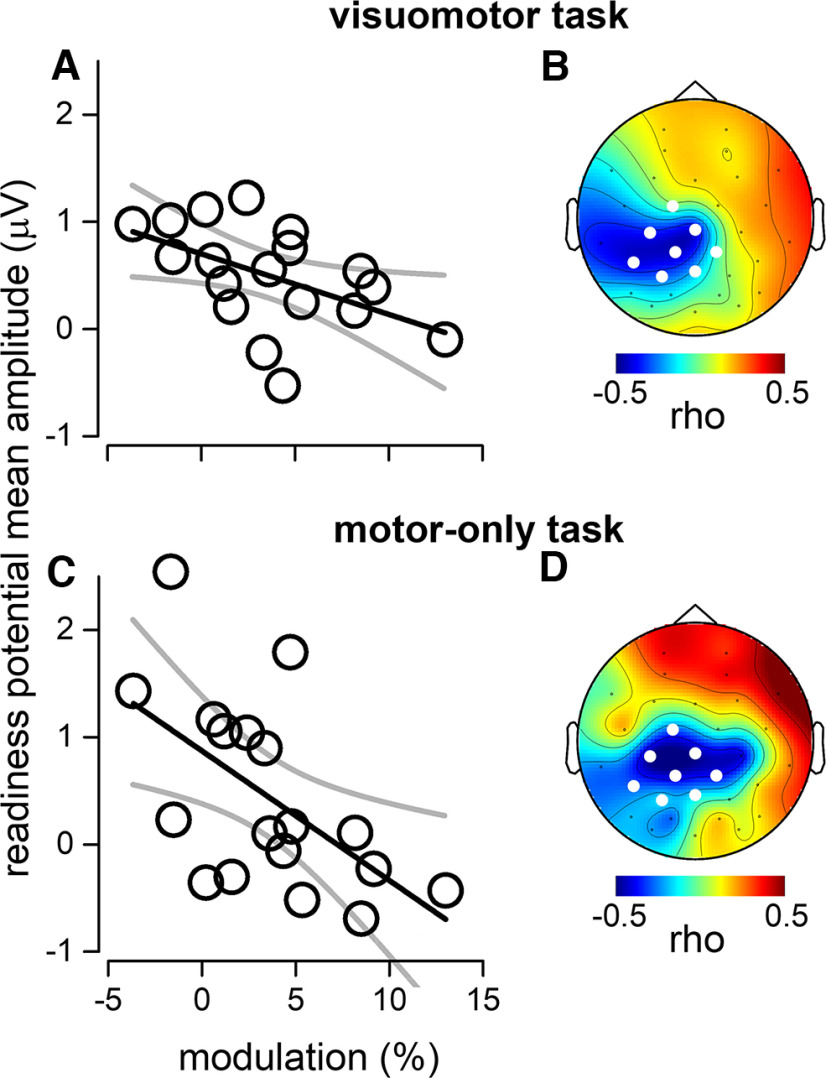
Results of correlational analyses. ***A***, Correlation between readiness potential and motor-induced modulation of visual perception across subjects in the visuomotor task (i.e., with visual stimulus). The magnitude of the perceptual modulation is plotted along the *x*-axis, and amplitude of the readiness potential averaged across the electrodes of interest (computed within the time interval from −500 to −100 ms relative to motor action) along the *y*-axis. The black solid line represents the best fit of the linear regression analysis and its 95% confidence bands. ***B***, The topographic map of the Pearson’s correlation coefficient calculated for each electrode; the electrodes of interest are highlighted in white: FC1, C3, CZ, CP5, CP1, CP2, P3, and PZ. See Extended Data Figure 2-1 for single channel correlation results. ***C***, Same as in ***A***, but the amplitude of the readiness potential was estimated during the motor-only task (i.e., without visual stimulus). The correlation between this response and the magnitude of the modulation in visual accuracy around the time of action execution (from the visuomotor task) was also significant (*p* < 0.05). ***D***, Same as in ***B*** during the motor-only task. In addition, analyses on the visual-evoked responses are reported in Extended Data Figure 2-2.

10.1523/ENEURO.0085-22.2022.f1-2 Extended Data Figure 1-2 Results of ICA for blinks. ***A***, Visuomotor condition. Top panel, Yellow lines mark blink occurrences measured for each individual trial. Each row plots a trial concatenating all participants’ data, *x*-axis shows the time from action execution. Bottom panel, Percentage of blink occurrence as a function of time from button press. The topographic plot shows the average scalp distribution of weight from the IC related to blinks. ***B***, Same as in ***A*** but for the motor-only condition. Download Figure 1-2, TIF file.

10.1523/ENEURO.0085-22.2022.f1-3 Extended Data Figure 1-3 Average percentage of blink occurrence as a function of time from button press for the five participants performing the visuomotor task while simultaneously monitoring gaze position (note that these recordings were performed as a separate test, as eye movements were not recorded during the main EEG experiment). The percentage and the distribution of blinks is comparable to that estimated with the ICA analysis. This suggests that motor-induced suppression was not caused by an increase rate of blinks around the time of button press. No saccades were detected around the time of button press, indicating that participants accurately followed the experimental instructions to maintain fixation. Download Figure 1-3, TIF file.

10.1523/ENEURO.0085-22.2022.f1-4 Extended Data Figure 1-4 Topography showing the slope of the ERPs in the motor-only condition, estimated with a linear regression analysis (see Materials and Methods) in the temporal window between –0.5 and –0.02 s. The topography of the effect is very similar to the visuomotor condition (compared to Fig. 1*B*). Download Figure 1-4, TIF file.

#### Motor-only task

In the motor-only task, participants simply had to press the button and look at the fixation point in the center of the screen. No visual stimulus was presented in this condition and, therefore, no response was required. As in the visuomotor condition, we asked participants to wait at least 1.5 s between successive button presses (mean and SD of interbutton-press interval: 2.75 ± 1.01 s).

### Data analysis

The EEG data were referenced to a common average and high pass filtered with a cutoff of 0.2 Hz (Blackman sinc FIR filter with a transition bandwidth of 0.4 Hz and filter order of 6876) using the MATLAB toolbox EEGLAB in combination with the plugin firfilt. Trials were epoched relative to the button press from −0.5 to 0.2 s. The ERPs were low-pass filtered at 40 Hz with an IIR Butterworth filter from the MATLAB toolbox Fieldtrip. As our primary interest was in the preaction activity, we defined an EEG baseline centered at action-onset (−0.05–0.05 s from keypress). In this condition, the readiness potential typically starts from a positive voltage and reaches 0 at the time of button press. Almost identical waveforms and topographies are obtained when using an earlier baseline (−0.5 to −0.4 s; Extended Data Figs. 1-1 and 1-4). As participants were prone to blink at a high frequency during the interval from −1 to −0.5 s from button press (see next paragraph), EEG activity >0.5 s before action onset was not suited for baseline correction.

To identify and exclude trials with blinks, ICA was run on the continuous high-pass filtered data (infomax ICA algorithm; Bell and Sejnowski, 1995) with a cutoff of 1 Hz (Blackman sinc FIR filter with a transition bandwidth of 2 Hz). After visually identifying and extracting the component related to blinks following standard criteria (left-right symmetry, frontal topography), we calculated the *z* score of the blink-related component, for each trial, in the interval −1–0.2 s. As eyelid-induced artifacts typically last for 200 ms (Plochl et al., 2012), trials with peaks of activity above 2 *z* scores within −0.7 and 0.1 s from button press were excluded from further analysis. The average percentage of trials excluded was 7.6% and 9.5% in the visuomotor and motor-only tasks respectively (see Extended Data Fig. 1-2). To overcome this limitation, we also verified fixation in 5 participants by measuring eye movement with an Eyelink 1000 (SR Research; see Extended Data Fig. 1-3 for results).

10.1523/ENEURO.0085-22.2022.f2-1 Extended Data Figure 2-1 Single channel correlation. ***A***, Topographic map of the Pearson’s correlation coefficient between the readiness potential amplitude and the motor-induced modulation of visual accuracy, calculated for each single electrode; electrodes reaching significant correlations are marked in white (*p* > 0.05, uncorrected). For the motor-only condition (left) a cluster of centro-parietal electrodes (C3, CZ, CP1, CP2, PO3) negatively correlated with the magnitude of the behavioral modulation, while only one frontal electrode (F8) positively correlated with it. For the visuomotor condition (right), a centro-posterior electrode (CP1) negatively correlated with the magnitude of the perceptual modulation, no other channels reached statistical significance. ***B***, Topographic map of the ERP grand-average scalp distribution in the time window between –0.5 and –0.1 s from button press, for the motor-only (left) and visuomotor tasks (right). The maps reveal the presence of an oriented dipole, with a negativity over front-temporal electrodes, right hemisphere. The grand-average ERPs computed at the electrodes FC1 (the focus of the positive activation over the left hemisphere) and F8 (the focus of the negative activation over the right hemisphere) are significantly anticorrelated (*p* < 0.001), suggesting the presence of an oriented dipole in the EEG signal driving the opposite correlation emerging in panel ***A***. Download Figure 2-1, TIF file.

10.1523/ENEURO.0085-22.2022.f2-2 Extended Data Figure 2-2 Analyses on the visual-evoked potentials. ***A***, Grand-average of ERPs for short (orange) and long ASIs trials (light blue) at the electrode PO4, for trials presented to the left visual field. ***B***, Same as in ***A*** but for trials presented to the right visual field, at PO3. Colored shaded areas indicate the standard error. For each participant, the average mean voltage of the VEP was computed over the parietal-occipital electrode contralateral to the side of visual stimulation (i.e., PO4/PO3 for stimuli presented on the left/right visual field, respectively), after removing 100 ms of prestimulus baseline. Contralateral left and right responses were then pooled together. A two-tail paired *t* test, comparing short and long ASIs, was run for each datapoint, and *p*-values were FDR corrected (q = 0.05). A significant difference emerged between the two conditions at around 250–270 ms (uncorrected *p* = 0.04); however, it did not survive FDR correction (pFDR > 0.05). A closer look at the topology of the EEG activity in that temporal window revealed the presence of nonlateralized, central, positive component peaking at around 250 ms, and mostly expressed over CZ (data not shown). Despite the interesting and preliminary finding at 250 ms, the primary components associated with early visual responses are not modulated differently as a function of the ASI from action onset. Finally, we pulled together the two ASIs to compare the ERPs of correct versus incorrect trials. No difference was found between the two VEPs (*p* > 0.05, uncorrected; data not shown). Given that the VEP response is elicited by the simultaneous presentation of high-contrast gratings in both the upper and lower visual field stimuli, it is likely that the elicited responses are being saturated, making it difficult to measure a modulation between late short and long ASI stimuli. Several papers reported a reduction in certain visual evoked components (namely, N1 and P2; Schafer and Marcus, 1973; Gentsch and Schütz-Bosbach, 2011; Hughes and Waszak, 2014; Mifsud et al., 2018), while other studies reported an amplification of other visual-evoked components (N145 and P1; Hughes and Waszak, 2011; Mifsud et al., 2016). This discrepancy has been mainly attributed to a diverse range of stimuli, experimental conditions, and preprocessing (Mifsud et al., 2018). Download Figure 2-2, TIF file.

Our analyses focused on readiness potential activity. We determined the eight electrodes that recorded the strongest readiness potential activity (i.e., stronger negative deflection) by evaluating the slope of a linear regression of the grand-average ERPs in the interval −0.5 to −0.02 s from button press in the visuomotor condition. Electrodes FC1, C3, CZ, CP5, CP1, CP2, P3, and PZ were selected given the strongest negative slope (mean ± SD: −2.02 ± 0.84 μV/s; see the topographic plot in Fig. 1*B*). Applying the same procedure, the same electrodes were selected for the motor-only condition (see Extended Data Fig. 1-4). Previous studies confirm that these electrodes are the ones typically expressing the strongest readiness potential response (Neshige et al., 1988; Ikeda et al., 1992; Shibasaki and Hallett, 2006; Di Russo et al., 2017).

To estimate modulation of perception around the time of action execution, trials from the visuomotor task were divided into two datasets: short and long action-stimulus intervals (ASIs). The short ASIs condition included trials in which the visual stimulus appeared no later than 120 ms after the button press; the long ASIs condition included trials with the visual stimulus occurring later than 600 ms after the button press. An exponential fit to the data confirmed a decrease in visual accuracy for ASIs close to the action, with accuracy reaching asymptote around 100–150 ms after the button press (*F*_(3,15)_ = 14,138.6; *p* < 0.001; Fig. 1*C*). The modulation of visual accuracy was estimated as the difference between the average accuracy in long versus short ASIs trials. The mean (±1 SD) number of trials across participants was 123 ± 11 and 129 ± 13 for short and long ASIs, respectively; for the two participants with more sessions, the mean number of trials was 318 ± 5 and 351 ± 9 for short and long ASIs, respectively. The analysis of visual response accuracy was repeated using only trials in which the stimulus occurred <100 ms after the button press in the short condition.

To estimate the amplitude of the readiness potential, we computed mean amplitudes over the electrodes of interest for the period −500 to −100 ms relative to button press (see Fig. 1*B*). These estimations were done separately for the visuomotor task and motor-only task. To test how the amplitude of the readiness potential relates to the magnitude of the modulation in performance, we computed the Pearson’s correlation coefficient across subjects (see also Extended Data Fig. 2-2 for a description of the visual-evoked potential results and methods, in the two conditions).

## Results

Eighteen volunteers were asked to indicate which grating (upper or lower) had the higher spatial frequency when two brief stimuli were presented randomly in either the left or the right visual hemifield, with 18 possible delays from action execution (ranging from 16 to 816 ms). Participants performed the task with an overall accuracy of 74 ± 2% (mean and standard error), all within 60–90% of accuracy. For each participant, modulation of visual accuracy was estimated as the difference between the average perceptual accuracy for stimuli presented far away from the action (long ASIs, with visuomotor delays >600 ms) and those presented close to the button press (short ASIs, with visuomotor delays <120 ms). Overall, long ASIs accuracy was higher than the short ASIs one, with an average improvement of ∼5% (Fig. 1*D*). A two-tailed paired-sample *t* test confirmed that visual accuracy was higher for long than for short ASIs (*t*_(17)_ = 3.55, *p* = 0.002). To assess whether this perceptual modulation was related to the position of the visual stimulus (left or right visual field), we split the dataset into stimuli presented to the left and stimuli presented to the right hemifield and contrasted the size of the modulation effects. The effect was not significantly different for stimuli presented on the left and right visual field (*t*_(17)_ = 1.08, *p* = 0.292), suggesting that the modulation effect was independent of the hemifield in which the stimulus occurred.

To be able to test for a correlation between the strength of the readiness potential and the modulation of visual perception, we computed the individual mean amplitudes of the readiness potential over the a-priori defined electrodes of interest: FC1, C3, CZ, CP5, CP1, CP2, P3, and PZ, for the temporal window −500 and −100 ms before the button press (Fig. 1*B*, light blue curve). We correlated the difference in visual accuracy for long and short ASIs with the amplitude of the readiness potential in the visuomotor task (Fig. 2*A*,*B*). The analysis revealed a negative correlation that was significant (*r*_(18)_ = −0.503, *p* = 0.033).

We also computed the individual mean amplitudes of the readiness potential in the motor-only task (Fig. 1*B*, orange curve). The readiness potential mean amplitudes in the motor-only condition were strongly correlated with the modulation of visual perception from the visuomotor task (*r*_(18)_ = −0.569, *p* = 0.013; Fig. 2*C*,*D*).

Previous studies on sensory attenuation and motor-induced suppression, have shown that those effects are generally reduced or almost abolished for sensorimotor delays larger than 100 ms (Blakemore et al., 1999; Aliu et al., 2009). To make our results more comparable to the existing literature, we replicated the correlation analyses by restricting the short ASI delays below 80 ms. Visual accuracy for these shorter ASIs was lower compared with long ASIs (*t*_(17)_ = 3.125; *p* = 0.006), and was significantly correlated with the amplitude of the readiness potential for both the visuomotor (*r*_(18)_ = −0.556; *p* = 0.016) and the motor only condition (*r*_(18)_ = −0.502; *p* = 0.033).

## Discussion

The characteristics of readiness potential, a slow EEG response that emerges during action preparation, has been associated with many functional differences that affect how action and perception interact over time (Reznik et al., 2018; Vercillo et al., 2018; Wen et al., 2018; Travers et al., 2021) and perceptual confidence (McAdam and Rubin, 1971). Despite this evidence, it is still unknown whether an association exists between this ERP component, indicative of motor preparation, and the effect of voluntary actions on visual perceptual accuracy. Here, we demonstrate that this motor-induced modulation of visual accuracy is associated with the readiness potential. Specifically, our findings show that the magnitude of this modulation correlates with the readiness potential amplitude in both visuomotor and motor-only tasks. This suggests that the processes underlying the readiness potential are linked to the modulation of visual perception around the time of action execution, and readiness potential may be a fingerprint of individual visuomotor interactions.

We found that discrimination accuracy for visual stimuli triggered by participants’ button press was significantly reduced for stimuli presented within the first 100 ms after the button press, as compared with when the visual stimuli occurred later in time (>600 ms). Given the difficulties of including a passive condition, balanced for sensory expectation and attentional load, we cannot establish whether the modulation is associated with peri-action performance suppression or, rather, postaction performance enhancement. However, previous findings have shown consistently reduced perceptual accuracy for visual stimuli triggered by voluntary hand movements compared with externally triggered stimuli (Cardoso-Leite et al., 2010; Stenner et al., 2014a; Vasser et al., 2019), suggesting a suppression of performance in our experiment as well. Interestingly, the temporal dynamic of the current modulation mimics the known dynamic of sensory attenuation in the auditory (Aliu et al., 2009) and tactile domains (Blakemore et al., 1999).

We estimated the readiness potential mean amplitudes for each subject within a time window of 500–100 ms before the button press and obtained an individual index of the motor-induced modulation of visual perception by computing the difference in accuracy between the short and long ASIs trials. The correlation between these two measures showed higher visual sensitivity around the time of action execution in participants with larger readiness potential amplitudes. Although earlier studies have related the readiness potential to confidence (McAdam and Rubin, 1971) and sensory anticipation following a self-initiated movement (Reznik et al., 2018; Vercillo et al., 2018; Wen et al., 2018; Travers et al., 2021), ours is the first study to implicate the readiness potential directly in visual sensitivity. McAdam and Rubin (1971) reported that the amplitude of the readiness potential is associated with confidence in perception of a visual stimulus presented right after the depression of the switch. They found that when participants were certain about the visual percept, their readiness potential was more negative then when they were doubtful or ambivalent about it. Their results were limited to visual stimuli presented with a fixed and predictable delay after the action, and the reported association might be mediated by cognitive processes and decision mechanisms. Our result shows that sensitivity, a signature of early visual processes, is associated with the amplitude of the readiness potential preceding the action and, more importantly, interindividual differences in readiness potential amplitudes recorded in a condition without visual stimulation are predictive of the magnitude of the individual perceptual modulation. This suggests that the amplitude of the readiness potential response is associated with a modulation in visual sensitivity around the time of action execution, and it predicts, even in the absence of visual stimuli and tasks, the magnitude of this modulation within each participant. This is consistent with recent fMRI findings with a similar task design, showing that primary visual cortex is rhythmically suppressed as a function of visual stimuli ASI from action onset (Benedetto et al., 2021).

Is the modulation of visual accuracy following a button press a form of motor-induced suppression, similar to that observed during saccadic eye movement (i.e., saccadic suppression)? The modulation reported here and saccadic suppression differ in at least one aspect. The magnitude of the modulation (∼5%) is not comparable to the suppression that is associated with saccadic eye movements. Saccadic suppression is much stronger, causing a complete phenomenological ablation of the visual input. Evidence suggests that saccadic suppression mostly derives from a selective suppression of the magnocellular visual pathway (Burr et al., 1994), mediated by a corollary discharge signal that changes the gain of the visual responses (Diamond et al., 2000; Ross et al., 2001; Binda and Morrone, 2018). The function of this suppression may be related to the selective suppression of the spurious motion signals generated by the eye movement (Burr et al., 1982). Whether the motor-induced suppression beyond the oculomotor system has the same function is less clear, as other types of movements (e.g., button press) may not induce similarly spurious visual signals. However, these movements can give rise to cross-modal interactions, and the small visual suppression we report may relate to the attenuation or recalibration of this cross-modal effect (Alais et al., 2010). For instance, saccades can affect auditory (Krüger et al., 2016; Gruters et al., 2018) and tactile (Harrar and Harris, 2009) perception. Similarly to our results, these cross-modal effects are less strong than the intramodal ones (Harris and Lieberman, 1996), leaving open the possibility that the modulation reported here may reflect a mechanism of motor-induced suppression.

Although the exact nature of readiness potential is still under debate (Schurger et al., 2021), a number of studies have shown that the readiness potential amplitude is modulated by sensory expectation (Reznik et al., 2018; Vercillo et al., 2018; Wen et al., 2018; Travers et al., 2021). It is well know that temporal expectation modulates visual performance over time (Nobre et al., 2007). For instance, Fiebelkorn et al. (2013) measured visual detection at several delays from the appearance of a visual cue. They found that detection rate increased with the cue-to-target delay. It has been argued that (pre)motor modulations may be confounded with attentional/anticipatory processes (Hughes et al., 2013; Stenner et al., 2014a, b). Although our study does not address this question, Stenner et al. (2014a) showed that visual accuracy, after controlling and accounting for stimulus predictability (i.e., sensory expectation) and motor output (i.e., motor prediction), was still reduced to self-generated visual stimuli. Interestingly, they also found enhanced prestimulus α activity (7.5–12.5 Hz) in the visual cortex when the identity and onset of the stimulus are controlled by participants’ motor actions. α Activity is typically associated with neuronal inhibition (Klimesch et al., 2007; Jensen et al., 2012), and prestimulus α has been shown to predict visual detection accuracy (Busch et al., 2009). Stenner and colleagues interpreted the prestimulus α activity in their study as a signature of sensory anticipation and attenuation induced by the movement (Stenner et al., 2014a).

The modulation of visual accuracy reported here might be influenced by (or reflect) a combination of different processes including motor actions, perception, and temporal predictions. Nevertheless, it is noteworthy that readiness potential in the motor-only task predicted the magnitude of the modulation of visual accuracy, although participants performed no visual task in that condition with no allocation of attention or visual expectation resources. Therefore, the correlation between the readiness potential and the differences in peri-action perception might also reflect the activity of temporal coordination of action and perception. This coordination is achieved by establishing a precise sensorimotor synchronization around the time of action execution This is consistent with evidence showing an association between the readiness potential dynamics and temporal recalibration of cortical activity after adaptation to altered visuo-motor temporal delays (Cai et al., 2018). Furthermore, the readiness potential has also been linked to intentional binding, which relates to the perceived time of sensory outcomes following a voluntary action (Jo et al., 2014).

Could this sensory-motor temporal coordination mechanism rely on efference copy signaling (Engel et al., 2001; Melloni et al., 2009)? Intriguingly, intentional binding is considered critical for developing a normal sense of agency, that is, the experience of controlling action to influence events in the environment (Moore and Obhi, 2012). Both sense of agency and intentional binding are thought to be impaired when efference copy signaling is dysfunctional, such as in schizophrenia and autism (Feinberg, 1978; Shergill et al., 2005; Ford et al., 2014; Yao et al., 2021). These impairments may be also associated with abnormal readiness potential amplitudes (Feinberg, 1978; Ford et al., 2014; Yao et al., 2021) and sensory attenuation for self-triggered stimuli (Shergill et al., 2005). Given these associations, the correlation that emerged from the current study might provide an interesting tool for studying intentional binding and sense of agency in individuals with different personal traits (e.g., schizotypical and autistic).

Although controversial (Wilke and Lansing, 1973; Hazemann et al., 1978), biophysical factors related to the preparation of specific movements, such as motor coordination and force, may also modulate the readiness potential (Ford et al., 1972; Kutas and Donchin, 1974; Becker and Kristeva, 1980; Kristeva et al., 1990). As we did not record participants’ kinematics, we cannot exclude the possibility that differences in the readiness potential amplitude across participants are because of differences in movement performance. Although visual suppression is known to increase with larger saccade and blink amplitudes (Volkmann et al., 1981; Stevenson et al., 1986), it is unclear whether this is also true for body movements, including the force of the button press. Therefore, the exact relationship of movement force to readiness potential amplitude and motor-induced visual suppression may need to be considered in future studies.

Our task required a fine visual discrimination which would have been strongly affected by failures to maintain central fixation and – in particular – by blinks (Volkmann, 1986). Therefore, the motor-induced modulation of visual perception we report here could also be explained by a difference in the probability of blink occurrences between the short and long ASIs conditions. However, our analysis suggests that blinks around the button press were rare (see Extended Data Figs. 1-2 and 1-3), implying that participants only tended to blink long after the stimulus presentation. Therefore, blinks are unlikely to account for the observed suppression in accuracy. Similarly, saccadic eye movements can also impact visual accuracy (Volkmann, 1986; Burr et al., 1994). However, in our paradigm, visual stimuli were randomly displayed on the left or right side of the monitor, which would favor central fixation as optimal strategy for better discrimination. We also used very brief stimuli that could have been easily suppressed during eye movement requiring, thus again, good fixation. Taken together, these observations suggest that the visual modulation reported in Figures 1 and 2 is related to the button press rather than eye movements.

In conclusion, visual sensitivity is modulated within a short time window around a voluntary action. Here, we showed that the readiness potential elicited by a button press correlates with this motor-induced modulation of visual perception, whether the action execution triggers a visual stimulus or not. This may suggest the presence of a general and automatic mechanism, possibly fundamental to establishing a precise visuomotor synchronization. Furthermore, we found that the readiness potential amplitude can predict the magnitude of individual modulation effects, which provides an interesting tool for studying normal functions and dysfunctions of visuomotor interactions in individual brains.

## Synthesis

Reviewing Editor: Nicholas J. Priebe, University of Texas at Austin Decisions are customarily a result of the Reviewing Editor and the peer reviewers coming together and discussing their recommendations until a consensus is reached. When revisions are invited, a fact-based synthesis statement explaining their decision and outlining what is needed to prepare a revision will be listed below. The following reviewer(s) agreed to reveal their identity: Thomas Whitford.

SYNTHESIS

Two reviewers evaluated the manuscript “The role of the readiness potential in motor-induced visual suppression” and both reviewers agree that the authors provide a compelling association between the readiness potential and motor-induced visual suppression. There was consensus, both in the reviews and in the consultations that followed, that there are issues that should be addressed to make the manuscript stronger. A major issue from raised in the manuscript is how efference copy is related to the readiness potential. Whereas efference copy may be linked to the readiness potential, evidence for this link is not provided in the current manuscript. Further the reviewers raised questions about the nomenclature used, the specific methods employed and commented on how this work is related to previous studies, particularly McAdams and Rubin (1971). These issues and others are detailed below, which you should find useful in revising your manuscript, if you decide to resubmit it to eNeuro or to another journal.

Major Issues:

1) Efference Copy: At prominent locations within the manuscript the result is interpreted as evidence for a link between the efference copy and motor-induced visual suppression (e.g., line 59, line 210). This conclusion is based on other papers that discussed the readiness potential as an indicator of the efference copy. However, this relation was not tested in the current study and there are alternative explanations for the origin of the readiness potential and for motor-induced sensory suppression. Moreover, a correlation might be mediated by processes less directly related to the measures. Thus, the results should be interpreted as evidence for an association between motor-induced suppression and the readiness potential and the efference copy should only be discussed as one possible mechanism behind both phenomena.

Typically, studies investigating ‘motor-induced suppression’ will compare an ‘active’ condition (in which the participant performs a movement to elicit a stimulus) to a ‘passive’ condition (in which the stimulus is not elicited by the participant’s movement). However, the present study did not include a ‘passive’ condition, which makes it somewhat difficult to place these results in the context of the existing literature. In the present study, ‘motor-induced suppression’ was defined by the difference in error rate between in trials in which the visual stimulus occurred ‘close to action’ (within 150 ms of the button-press) vs. trials where it occurred ‘far from action’ (over 600 ms from the button-press). In the absence of a ‘passive condition’, how can the authors be sure that this effect was a consequence of the movement?

There may be ‘low-level’ differences in attention between the two trial types - e.g., maybe if two events occur close in time then attention is split between them? Might the same effect occur when comparing trials that were ‘close to an external cue’ vs. ‘far from an external cue’?

2) This work appears to overlap substantially with the report from McAdam and Rubnin (1971), using different stimuli and a condition without visual stimulus presentation. It would be appropriate to comment on how the present work relates to that prior work and describe the additional knowledge that is gained from this study.

3) The title states that there might be a role for the readiness potential in motor-induced visual suppression, however, this implies causality which is not supported by the study. It would be more accurate to speak of an association or correlation throughout, including in the title.

Methodological Issues:

1) How were the verbal responses coded, by the experimenter? How did participants know that enough time had passed to respond or trigger the next stimulus? What happened if they responded too early?

2) The ICA identified a relatively high percentage of trials with eye artifacts. Is this due to a huge proportion of blinks? Does the ICA identify small eye movements? Is it simply oversensitive?

3) The naming ‘close-to-action’ and ‘far-from-action’ could be confusing as it implies a spatial dimension. Something more related to the temporal domain would be easier for the reader, for example, short stimulus delay or rapid stimulus onset. Further, defining ‘close-to-action’ as within 150 ms and ‘far-from-action’ as over 600 ms seems somewhat arbitrary - were there any a priori reasons to select these windows, or was the decision purely data-driven?

Some previous studies have found that inserting a delay of as little as 100 ms reduces abolishes the motor-induced suppression effect to auditory stimuli - do the authors have any thoughts on why this window would be different for visual stimuli?

It would be interesting to know if the ‘close to action’ stimuli elicited a smaller visual-evoked responses (i.e., post-stimulus) than the ‘far from action’ condition.

While the literature in motor-induced visual suppression is quite sparse, I note that a previous study actually found button-press-elicited visual stimuli to evoke *larger* visual-evoked responses than externally-generated visual stimuli (Mifsud et al., 2016, Psychophysiology, 53, 723-32).

4) Please report either p-values or Bayes factors, but not both. If Bayes factors are reported specify the contrasted hypotheses rather than frequentist test statistics (F- or t-values).

5) There are several passages that read as if the EEG signals at the selected electrodes are exclusively generated in motor areas. Given that these are EEG signals, that cannot be true. Please either change this phrasing or add source reconstruction.

6) The topographic maps in Figure 2 are confusing. Is the mean amplitude during the pre-action interval was averaged across the selected electrodes? The topography seems to suggest that correlations were calculated for each electrode. Further, the color code suggests a strong positive correlation at ipsilateral fronto-temporal electrodes. How can that be?

7) Regarding the readiness potential waveforms, it is a bit confusing as to why the RPs were positive voltage until one realizes that a baseline of -50 to +50 ms was used. This would have the effect of ‘pinning’ the waveforms together at the time of stimulus onset. Did the visuomotor waveforms and motor-only waveforms have similar shapes / topographies when using an earlier baseline? E.g., -1500 to 1000 ms?

Minor Issues:

line 32: motions -> motion signals

line 36: visual -> visually

line 40: It is not clear how the TMS result relates to visual cortex activity.

line 93: 3 dva to the left/right or up/down?

line 156: It is not clear at this point how motor-induced visual suppression was quantified, i.e., which measure was correlated with the mean amplitude? Probably, the correlation should just be described at the end of the next paragraph.

line 184: the statistical results do not confirm the absence of a difference, they fail to indicate the presence of a difference

line 218: ) , -> ),

line 220: from button press -> after the button press

line 225: Which argument is being made regarding the study by Stenner and colleagues?

Figure 1B: does the scalp heatmap show the slope of the ERP for all electrodes or just the selected ones? Edit: the methods suggest all electrodes, that should be indicated in the caption

Figure 1D: the axis labels should indicate somehow that the figure shows average accuracies

Figure 2A: The caption should indicate that the mean amplitude within the time interval from -500 to -100 ms relative to the motor action is shown.

## Author Response

Synthesis of Reviews:

Computational Neuroscience Model Code Accessibility Comments for Author (Required):

N/A

Synthesis Statement for Author (Required):

SYNTHESIS

Two reviewers evaluated the manuscript “The role of the readiness potential in motor-induced visual suppression” and both reviewers agree that the authors provide a compelling association between the readiness potential and motor-induced visual suppression. There was consensus, both in the reviews and in the consultations that followed, that there are issues that should be addressed to make the manuscript stronger. A major issue from raised in the manuscript is how efference copy is related to the readiness potential. Whereas efference copy may be linked to the readiness potential, evidence for this link is not provided in the current manuscript. Further the reviewers raised questions about the nomenclature used, the specific methods employed and commented on how this work is related to previous studies, particularly McAdams and Rubin (1971). These issues and others are detailed below, which you should find useful in revising your manuscript, if you decide to resubmit it to eNeuro or to another journal.

_______________________________________________________________________________________

Thanks to the editor and the referees, for the general positive assessment of our study and for providing constructive criticisms that helped us to improve the manuscript.

_______________________________________________________________________________________

Major Issues:

(1) Efference Copy: At prominent locations within the manuscript the result is interpreted as evidence for a link between the efference copy and motor-induced visual suppression (e.g., line 59, line 210). This conclusion is based on other papers that discussed the readiness potential as an indicator of the efference copy. However, this relation was not tested in the current study and there are alternative explanations for the origin of the readiness potential and for motor-induced sensory suppression. Moreover, a correlation might be mediated by processes less directly related to the measures. Thus, the results should be interpreted as evidence for an association between motor-induced suppression and the readiness potential and the efference copy should only be discussed as one possible mechanism behind both phenomena.

Typically, studies investigating ‘motor-induced suppression’ will compare an ‘active’ condition (in which the participant performs a movement to elicit a stimulus) to a ‘passive’ condition (in which the stimulus is not elicited by the participant’s movement). However, the present study did not include a ‘passive’ condition, which makes it somewhat difficult to place these results in the context of the existing literature.

_______________________________________________________________________________________

We thank the reviewer for pointing this out. We agree that our study provides only evidence for an association between readiness potential and motor-induced visual modulation. The links between efference copy and motor-induced visual modulation are speculative, and we have mitigated this claim in the current version.

We agree with the reviewer that ‘motor-induced suppression’ is traditionally measured as the difference between an active and a passive condition, which we could not record since we were interested in studying the temporal dynamic of sensitivity after an action. To avoid confusion with the existing literature of “motor-induced suppression", we now refer to our perceptual effect as a modulation of visual accuracy around the time of action execution. This modulation leads to a significant difference in visual accuracy for short vs long SOAs, but - as the reviewer pointed out - it can equally result from a peri-action suppression or from a post-action enhancement. We now address this issue in the discussion. Accordingly, we have changed the titled and the text.

_______________________________________________________________________________________

In the present study, ‘motor-induced suppression’ was defined by the difference in error rate between in trials in which the visual stimulus occurred ‘close to action’ (within 150 ms of the button-press) vs. trials where it occurred ‘far from action’ (over 600 ms from the button-press). In the absence of a ‘passive condition’, how can the authors be sure that this effect was a consequence of the movement? There may be ‘low-level’ differences in attention between the two trial types - e.g., maybe if two events occur close in time then attention is split between them? Might the same effect occur when comparing trials that were ‘close to an external cue’ vs. ‘far from an external cue’?

_______________________________________________________________________________________

Yes, and now we discuss the possible influence of splitting or deploying attention or of hazard time. However, the correlation between the readiness potential recorded during the ‘motor-only’ condition (i.e., no visual expectations/attention) and the modulation of visual performance for the different SOA suggests that the effect is not attentional but reflects the preparation of the movement.

It is known in the visual literature that hazard time and the time from attentional allocation generate a monotonic decrease or increase of performance with time (Nobre et al., 2007). For instance, Fiebelkorn et al. (2013) measured visual detection at several delays from the appearance of visual cue. Similar to our results, they found that detection rate increased with the delay between cue and target. It is therefore possible that these two phenomena rely on a similar (attentional) mechanism, in one case related to the visual cue, in our case related to the motor attention required for the button press. Interestingly, Fiebelkorn and colleagues proposed that this mechanism might reflect the involvement of a sensorimotor network, governed by rhythmic processes periodically reweighting functional connections between higher-order brain regions and either sensory or motor regions (Fiebelkorn and Kastner, 2019). We believe that our correlation data in absence of visual attentional allocation or hazard time (motor-only condition), reinforce the intuition that the modulation is linked to the activity in pre-motor motor area.

_______________________________________________________________________________________

(2) This work appears to overlap substantially with the report from McAdam and Rubin (1971), using different stimuli and a condition without visual stimulus presentation. It would be appropriate to comment on how the present work relates to that prior work and describe the additional knowledge that is gained from this study.

_______________________________________________________________________________________

Thanks, we have expanded the description of this historical paper. McAdam and Rubin (1971) reported the first evidence of a possible involvement of readiness potential in vision, but the experiment suffered from many possible confounds, these include: (i) authors investigated the link between perceptual confidence (not visual accuracy) and the amplitude of the readiness potential on average across the subject population demonstrating that the RP is stronger for high confidence subjective report; (ii) results are limited to visual stimuli presented at a fixed and predictable delay following the action and to a simple localization task. To our knowledge, our study is the first showing that (i) the magnitude of the motor-induced visual modulation of sensitivity (not confidence or bias) following a voluntary button press is associated with the amplitude of the readiness potential preceding the same action, and (ii) interindividual differences in readiness potential amplitudes recorded in a condition without visual stimulation predict the magnitude of the individual perceptual modulation.

_______________________________________________________________________________________

(3) The title states that there might be a role for the readiness potential in motor-induced visual suppression, however, this implies causality which is not supported by the study. It would be more accurate to speak of an association or correlation throughout, including in the title.

_______________________________________________________________________________________

Based on to the reviewer’s suggestions, we changed the title to: “The readiness potential correlates with action-linked modulation of visual accuracy” and reworded the ambiguous sentences throughout the manuscript (see also major point #1).

_______________________________________________________________________________________

Methodological Issues:

(1) How were the verbal responses coded, by the experimenter? How did participants know that enough time had passed to respond or trigger the next stimulus? What happened if they responded too early?

_______________________________________________________________________________________

Participants were instructed to wait at least 1.5 s from the stimulus onset before providing a verbal response, coded by the experimenter, and also waited at least another 1.5 s before starting the next trial. In case participants responded/started too early, the experimenter provided online feedback. Before starting the data recording, all participants were trained to maintain this slow timing. When they felt confident, we started the data acquisition. Participants waited on average 2.6{plus minus}1 s from stimulus onset before providing a verbal response. Occasionally (< 1 % of trials) they responded too quicky (< 1 s), but in order not to bias the sample, we decided to keep all trials. We now report this information in the Method section.

_______________________________________________________________________________________

(2) The ICA identified a relatively high percentage of trials with eye artifacts. Is this due to a huge proportion of blinks? Does the ICA identify small eye movements? Is it simply oversensitive?

_______________________________________________________________________________________

We carefully screened our data for eye movement artifacts (that could affect both behavioral and EEG results) by taking a conservative approach rather than a more liberal one. For this reason, we used a conservative threshold (2 z-score) to define blinks in the interval between -700 to 100 ms from button press. The relatively high proportion of trials containing blinks (around 8% of trials) is probably due to the long wait between trials: subjects likely tried to avoid blinking after the button press in order not to miss the short stimulus presentation. Small eye movements (e.g., microsaccades) couldn’t be detected via the ICA procedure. We now report this limitation in the manuscript. However, in our control analysis a subset of participants performed the task while we recorded eye-movements. This control revealed that pressing the button was not causing the emergence of (micro)saccades synchronized with the action.

_______________________________________________________________________________________

(3) The naming ‘close-to-action’ and ‘far-from-action’ could be confusing as it implies a spatial dimension. Something more related to the temporal domain would be easier for the reader, for example, short stimulus delay or rapid stimulus onset.

_______________________________________________________________________________________

Thanks for the suggestions. We now changed the label to “short” and “long” SOAs.

_______________________________________________________________________________________

Further, defining ‘close-to-action’ as within 150 ms and ‘far-from-action’ as over 600 ms seems somewhat arbitrary - were there any a priori reasons to select these windows, or was the decision purely data-driven?

_______________________________________________________________________________________

The existing literature shows sensory attenuation for stimuli presented up to about 100 ms from the execution of an action (e.g., Blakemore et al., 1999). In the previous version of the manuscript, we defined short-SOAs stimuli presented within 150 ms from action execution, but - due to the step size of our sampling - the actual cut-off was 116 ms (i.e., stimuli presented at 150 ms are not considered as short-SOAs). We clarify this aspect and report the actual delay. We also replicated the analyses of the manuscript defining “short” SOAs as delays <80 ms and we replicated all the findings. In particular, we found that accuracy for this shorter SOAs was lower as compared to long SOAs (t(17) = 3.125; p = 0.006), and we found a significant correlation between the amplitude of the readiness potential and the suppression for both the visuomotor (r(18) = -0.556; p = 0.016) and the motor only condition (r(18) = -0.502; p = 0.033). We now report this additional analysis in the manuscript.

_______________________________________________________________________________________

Some previous studies have found that inserting a delay of as little as 100 ms reduces abolishes the motor-induced suppression effect to auditory stimuli - do the authors have any thoughts on why this window would be different for visual stimuli?

_______________________________________________________________________________________

As mentioned in the previous comment, the bound to define “short” SOAs is 116 ms, very close to the 100 ms windows mentioned by the reviewer. Our data shows that the motor-induced modulation is mostly abolished for the 150 ms delay, similarly to what reported in the sensory attenuation literature for the auditory (Aliu et al., 2009) and tactile domains (Blakemore et al., 1999).

_______________________________________________________________________________________

It would be interesting to know if the ‘close to action’ stimuli elicited a smaller visual-evoked responses (i.e., post-stimulus) than the ‘far from action’ condition. While the literature in motor-induced visual suppression is quite sparse, I note that a previous study actually found button-press-elicited visual stimuli to evoke *larger* visual-evoked responses than externally-generated visual stimuli (Mifsud et al., 2016, Psychophysiology, 53, 723-32).

_______________________________________________________________________________________

EEG studies on sensory attenuation in the visual domain are few and report mixed results. For instance, some studies reported a reduction in certain evoked components (namely N1 and P2) (Schafer and Marcus, 1973; Gentsch and Schütz-Bosbach, 2011; Hughes and Waszak, 2014; Mifsud et al., 2018), while other studies have reported an amplification of other visual evoked components (N145 and P1) (Hughes and Waszak, 2011; Mifsud et al., 2016). This discrepancy has been mainly attributed to a diverse range of stimuli and preprocessing (Mifsud et al., 2018). We now include analyses on the visual evoked potential response in the Extended Data. The VEP response in PO4 and PO3 is elicited by the simultaneously presentation of high-contrast gratings in both the upper and lower visual field stimuli. The VEP response is likely saturated, which makes it difficult to measure a modulation between short and long SOA stimuli. We also found that VEPs did not differ between correct and incorrect trials. We now report these results in the Extended Data. The difference in the ERPs observed around 250 ms after the stimulus onset reached statistical significance but did not survive FDR correction. A closer look at the topology of the EEG activity in that temporal window, revealed presence of non-lateralized, central, positive component, peaking at around 250 ms, and mostly expressed over Cz (not shown). However, the primary components associated to early visual responses are not modulated differently as function of the SOA from action onset.

Extended Data Figure 2-2. Analyses on the visual evoked potentials. (A) Grand-average of ERPs for short (orange) and long SOAs trials (light blue) at the electrode PO4, for trials presented to the left visual field. (B) Same as in (A) but for trials presented to the right visual field, at PO3.

_______________________________________________________________________________________

(4) Please report either p-values or Bayes factors, but not both. If Bayes factors are reported specify the contrasted hypotheses rather than frequentist test statistics (F- or t-values).

_______________________________________________________________________________________

Thanks, we removed the BF analysis, reporting only the more traditional frequentist approach.

_______________________________________________________________________________________

(5) There are several passages that read as if the EEG signals at the selected electrodes are exclusively generated in motor areas. Given that these are EEG signals, that cannot be true. Please either change this phrasing or add source reconstruction.

_______________________________________________________________________________________

We thank the reviewer for this comment. The relatively low number of electrodes (32) did not allow source reconstruction analysis (Michel et al., 2004; Michel and Brunet, 2019). For this reason, we rephrased those sentences.

_______________________________________________________________________________________

(6) The topographic maps in Figure 2 are confusing. Is the mean amplitude during the pre-action interval was averaged across the selected electrodes? The topography seems to suggest that correlations were calculated for each electrode. Further, the color code suggests a strong positive correlation at ipsilateral fronto-temporal electrodes. How can that be?

_______________________________________________________________________________________

We apologize for the confusion. The amplitude during the pre-action interval was calculated on the average if all the selected electrodes (marked in white over the head). For completeness, we also show in the topography maps the correlations computed for each single electrode. This map confirms the presence of a strong negative correlation that overlaps the topography maps in figure 1b (and extended data figure 1-3) showing the topology of the readiness-potential response. We now clarify this aspect in the manuscript.

The topoplot below shows the average ERP scalp distribution in the time window between -0.5 and -0.1 s from button press, for the visuomotor (left) and motor-only tasks (right). The plot reveals the presence of an oriented dipole, with a negativity over fronto-temporal electrodes, right hemisphere.

The grand-average ERPs computed at the electrodes FC1 (the focus of the positive activation over the left hemisphere) and F8 (the focus of the negative activation over the right hemisphere) are significantly anticorrelated (p<0.001), suggesting the presence of an oriented dipole in the EEG signal driving the opposite correlation we see in figure 2. We investigate this aspect further by looking at the p-values (uncorrected) of the single-channel correlations shown in figure 2B&D. For the visuomotor condition, a centro-posterior electrode (CP1, white electrode on the left topoplot here below) negatively correlated with the magnitude of the motor-induced visual modulation, no positive correlations reached statistical significance; for the motor-only condition a cluster of centro-parietal electrodes (C3, CZ, CP1, CP2, PO3, white electrodes on the right topoplot here below) negatively correlated with the magnitude of the behavioral modulation, while only one frontal electrode (F8, white electrode on the right topoplot here below) positively correlated with it.

We now included these analyses in the extended data.

_______________________________________________________________________________________

(7) Regarding the readiness potential waveforms, it is a bit confusing as to why the RPs were positive voltage until one realizes that a baseline of -50 to +50 ms was used. This would have the effect of ‘pinning’ the waveforms together at the time of stimulus onset. Did the visuomotor waveforms and motor-only waveforms have similar shapes / topographies when using an earlier baseline? E.g., -1500 to 1000 ms?

_______________________________________________________________________________________

Below we show the readiness potential curve after using a baseline correction from -0.5 to -0.4 s, as well as the corresponding intensity map of ERP slope in the interval -0.5 and -0.02 s from the keypress, for all electrodes. The waveforms and topographies are very similar to the ones reported in the manuscript (figure 1). In the main paper, we were interested in looking at the pre-action activity, therefore a baseline centered at action-onset (as the one we adopted) was ideal. As we screened the dataset for eye movements in the temporal proximity of the button press (see also methodological issue #2), we cannot safely use baselines before 500 ms.

_______________________________________________________________________________________

Minor Issues:

line 32: motions -> motion signals

line 36: visual -> visually

line 218: ) , -> ),

line 220: from button press -> after the button press

_______________________________________________________________________________________

Corrected.

_______________________________________________________________________________________

line 40: It is not clear how the TMS result relates to visual cortex activity.

_______________________________________________________________________________________

We rephrased the sentence to clarify the importance of these TMS results in our study.

_______________________________________________________________________________________

line 93: 3 dva to the left/right or up/down?

_______________________________________________________________________________________

Corrected, it was left/right.

_______________________________________________________________________________________

line 156: It is not clear at this point how motor-induced visual suppression was quantified, i.e., which measure was correlated with the mean amplitude? Probably, the correlation should just be described at the end of the next paragraph.

_______________________________________________________________________________________

We moved the paragraph as suggested by the reviewer.

_______________________________________________________________________________________

line 184: the statistical results do not confirm the absence of a difference, they fail to indicate the presence of a difference

_______________________________________________________________________________________

Corrected.

_______________________________________________________________________________________

line 225: Which argument is being made regarding the study by Stenner and colleagues?

_______________________________________________________________________________________

We clarified our argument. Stenner et al. (2014) showed that accuracy - after controlling and accounting for stimulus predictability (i.e., sensory expectation) and motor output (i.e., motor prediction) - was still reduced to self-generated visual stimuli. Interestingly, they also found enhanced pre-stimulus alpha activity (7.5-12.5 Hz) in the visual cortex when the identity and onset of the stimulus are controlled by participants’ motor actions. Alpha activity is typically associated with neuronal inhibition (Klimesch et al., 2007; Jensen et al., 2012), and pre-stimulus alpha has been shown to predict visual detection accuracy (Busch et al., 2009). Stenner and colleagues interpreted the pre-stimulus alpha activity in their study as a signature of motor-induced sensory anticipation and attenuation (Stenner et al., 2014)

_______________________________________________________________________________________

Figure 1B: does the scalp heatmap show the slope of the ERP for all electrodes or just the selected ones? Edit: the methods suggest all electrodes, that should be indicated in the caption

_______________________________________________________________________________________

We clarified the sentence and added the info in the caption.

_______________________________________________________________________________________

Figure 1D: the axis labels should indicate somehow that the figure shows average accuracies

_______________________________________________________________________________________

Corrected.

_______________________________________________________________________________________

Figure 2A: The caption should indicate that the mean amplitude within the time interval from -500 to -100 ms relative to the motor action is shown.

_______________________________________________________________________________________

Corrected.

_______________________________________________________________________________________

Bibliography:

Aliu SO, Houde JF, Nagarajan SS (2009) Motor-induced suppression of the auditory cortex. J Cognitive Neurosci 21:791-802.

Blakemore S-J, Frith CD, Wolpert DM (1999) Spatio-temporal prediction modulates the perception of self-produced stimuli. J Cognitive Neurosci 11:551-559.

Busch NA, Dubois J, VanRullen R (2009) The Phase of Ongoing EEG Oscillations Predicts Visual Perception. Journal of Neuroscience 29:7869-7876.

Fiebelkorn IC, Kastner S (2019) A Rhythmic Theory of Attention. Trends Cogn Sci 23:87-101.

Fiebelkorn IC, Saalmann YB, Kastner S (2013) Rhythmic sampling within and between objects despite sustained attention at a cued location. Current biology 23:2553-2558.

Gentsch A, Schütz-Bosbach S (2011) I did it: unconscious expectation of sensory consequences modulates the experience of self-agency and its functional signature. J Cognitive Neurosci 23:3817-3828.

Hughes G, Waszak F (2011) ERP correlates of action effect prediction and visual sensory attenuation in voluntary action. Neuroimage 56:1632-1640.

Hughes G, Waszak F (2014) Predicting faces and houses: Category-specific visual action-effect prediction modulates late stages of sensory processing. Neuropsychologia 61:11-18.

Jensen O, Bonnefond M, VanRullen R (2012) An oscillatory mechanism for prioritizing salient unattended stimuli. Trends in Cognitive Sciences 16:200-206.

Klimesch W, Sauseng P, Hanslmayr S (2007) EEG alpha oscillations: The inhibition-timing hypothesis. Brain Res Rev 53:63-88.

McAdam DW, Rubin EH (1971) Readiness potential, vertex positive wave, contingent negative variation and accuracy of perception. Electroencephalogr Clin Neurophysiol 30:511-517.

Michel CM, Brunet D (2019) EEG source imaging: a practical review of the analysis steps. Frontiers in neurology 10:325.

Michel CM, Murray MM, Lantz G, Gonzalez S, Spinelli L, De Peralta RG (2004) EEG source imaging. Clinical neurophysiology 115:2195-2222.

Mifsud NG, Beesley T, Watson TL, Whitford TJ (2016) Attenuation of auditory evoked potentials for hand and eye-initiated sounds. Biological psychology 120:61-68.

Mifsud NG, Beesley T, Watson TL, Elijah RB, Sharp TS, Whitford TJ (2018) Attenuation of visual evoked responses to hand and saccade-initiated flashes. Cognition 179:14-22.

Nobre AC, Correa A, Coull JT (2007) The hazards of time. Current opinion in neurobiology 17:465-470.

Schafer EW, Marcus MM (1973) Self-stimulation alters human sensory brain responses. Science 181:175-177.

Stenner MP, Bauer M, Haggard P, Heinze HJ, Dolan R (2014) Enhanced Alpha-oscillations in Visual Cortex during Anticipation of Self-generated Visual Stimulation. J Cognitive Neurosci 26:2540-2551.
