# Supplementary figures and images for: The Readiness Potential Correlates with Action-Linked Modulation of Visual Accuracy
Source: eNeuro. 2022 Nov 22;9(6):ENEURO.0085-22.2022. doi: 10.1523/ENEURO.0085-22.2022 (PMC9698660; doi:10.1523/ENEURO.0085-22.2022)

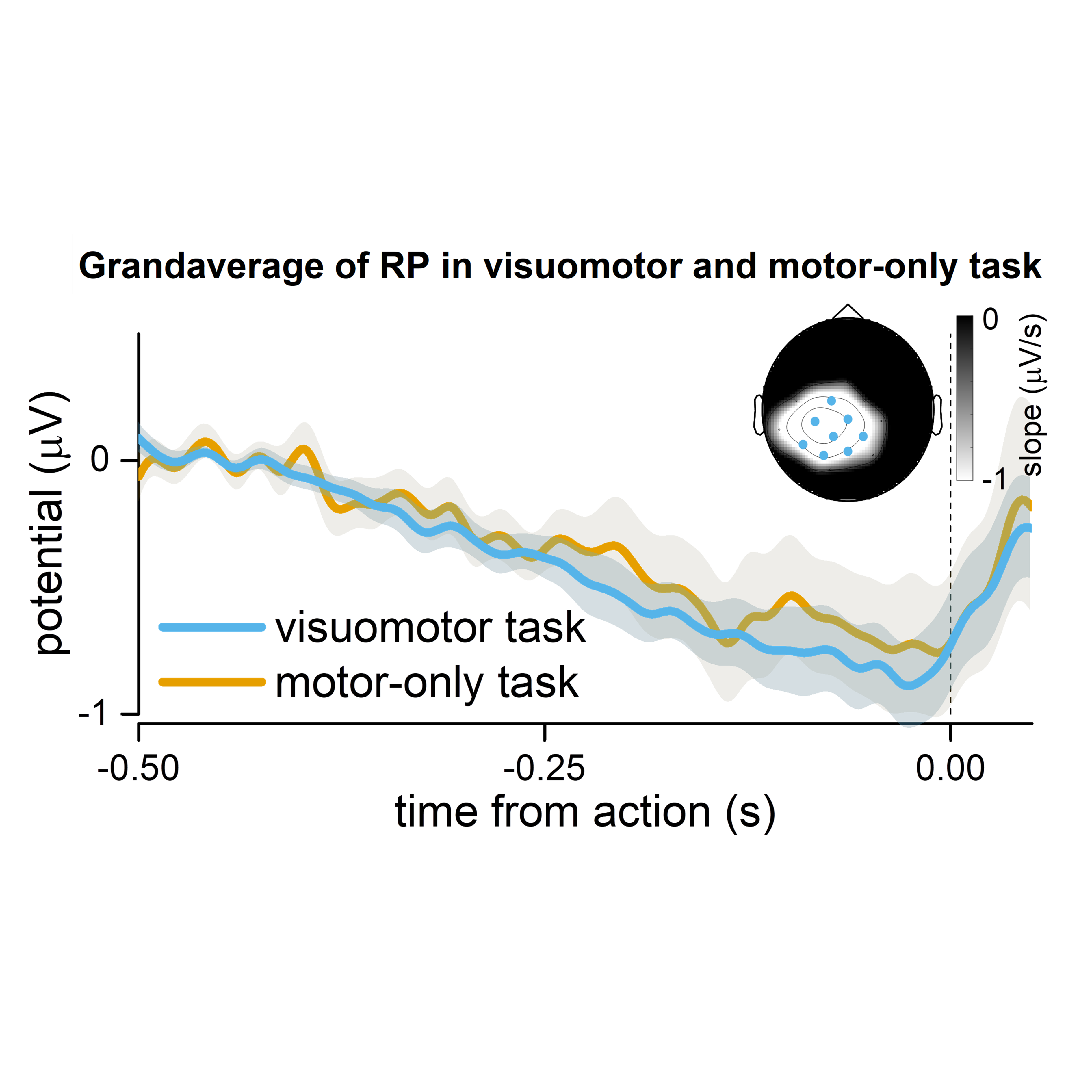

Supplement: Extended Data Figure 1-1 — Readiness potential activity with baseline computed between −0.5 and 0.4 s. A, Time course of readiness potential relative to the button press. The light blue and orange lines show the grand-averaged ERPs in the visuomotor and motor-only tasks, respectively, relative to action onset (0 s). Colored shaded areas indicate the standard error. The ERPs reflect the average activity at eight electrodes of interest: FC1, C3, CZ, CP5, CP1, CP2, P3, highlighted in blue in the inset. The intensity map of the topographical EEG plot shows the ERP slope in the interval −0.5 and −0.02 s from the keypress, for all electrodes. Download Figure 1-1, TIF file. [file enu-eN-NWR-0085-22-s02.tif]

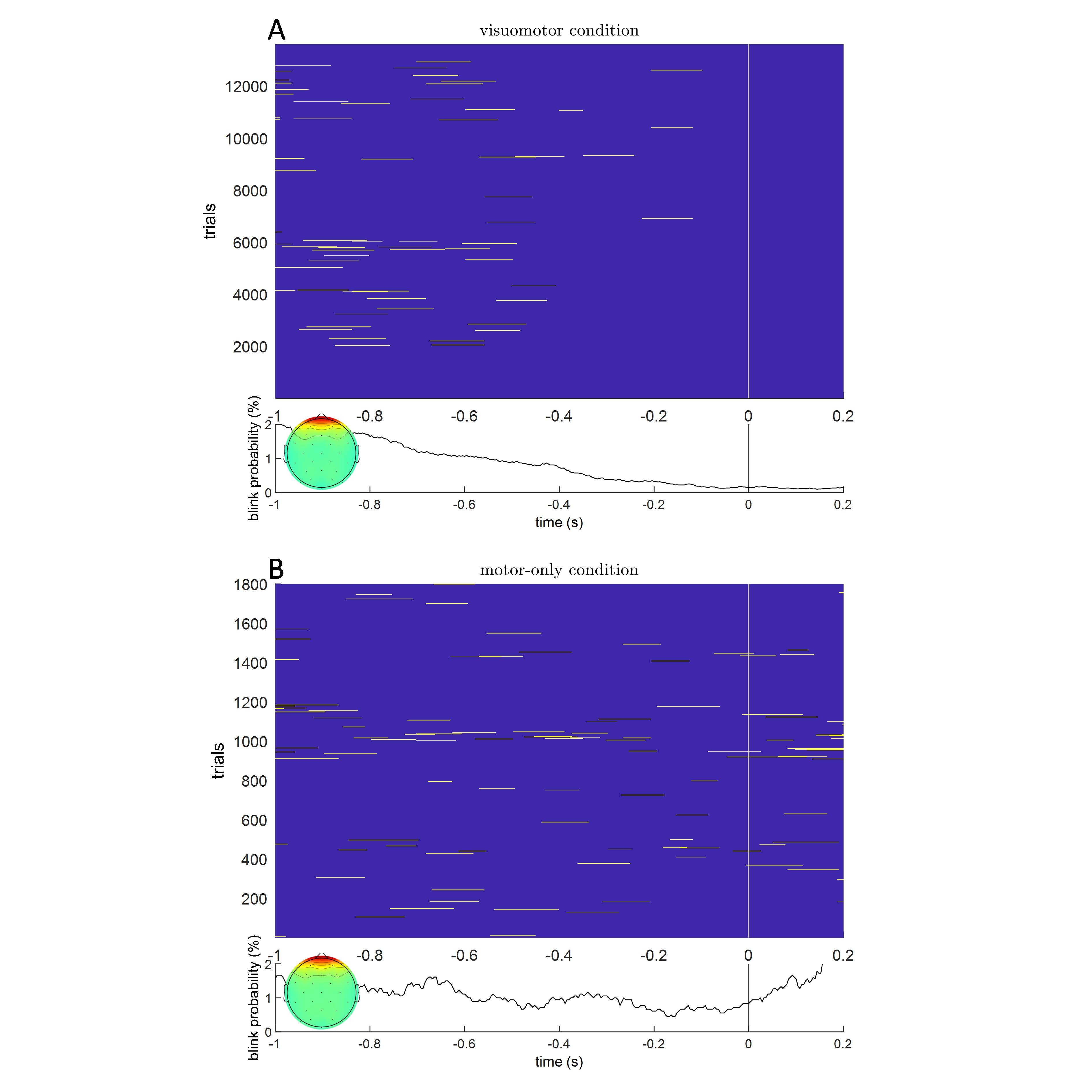

Supplement: Extended Data Figure 1-2 — Results of ICA for blinks. A, Visuomotor condition. Top panel, Yellow lines mark blink occurrences measured for each individual trial. Each row plots a trial concatenating all participants’ data, x-axis shows the time from action execution. Bottom panel, Percentage of blink occurrence as a function of time from button press. The topographic plot shows the average scalp distribution of weight from the IC related to blinks. B, Same as in A but for the motor-only condition. Download Figure 1-2, TIF file. [file enu-eN-NWR-0085-22-s03.tif]

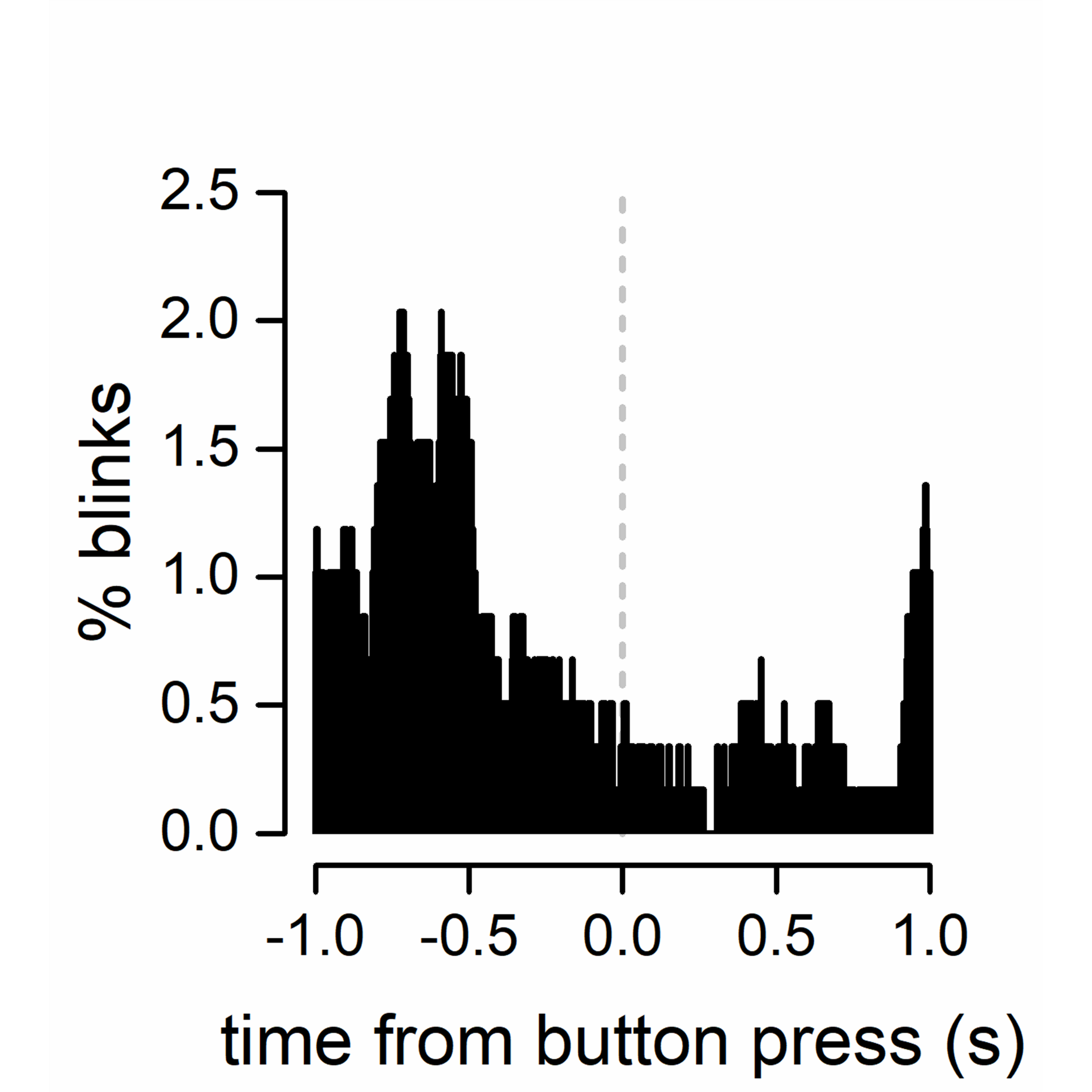

Supplement: Extended Data Figure 1-3 — Average percentage of blink occurrence as a function of time from button press for the five participants performing the visuomotor task while simultaneously monitoring gaze position (note that these recordings were performed as a separate test, as eye movements were not recorded during the main EEG experiment). The percentage and the distribution of blinks is comparable to that estimated with the ICA analysis. This suggests that motor-induced suppression was not caused by an increase rate of blinks around the time of button press. No saccades were detected around the time of button press, indicating that participants accurately followed the experimental instructions to maintain fixation. Download Figure 1-3, TIF file. [file enu-eN-NWR-0085-22-s04.tif]

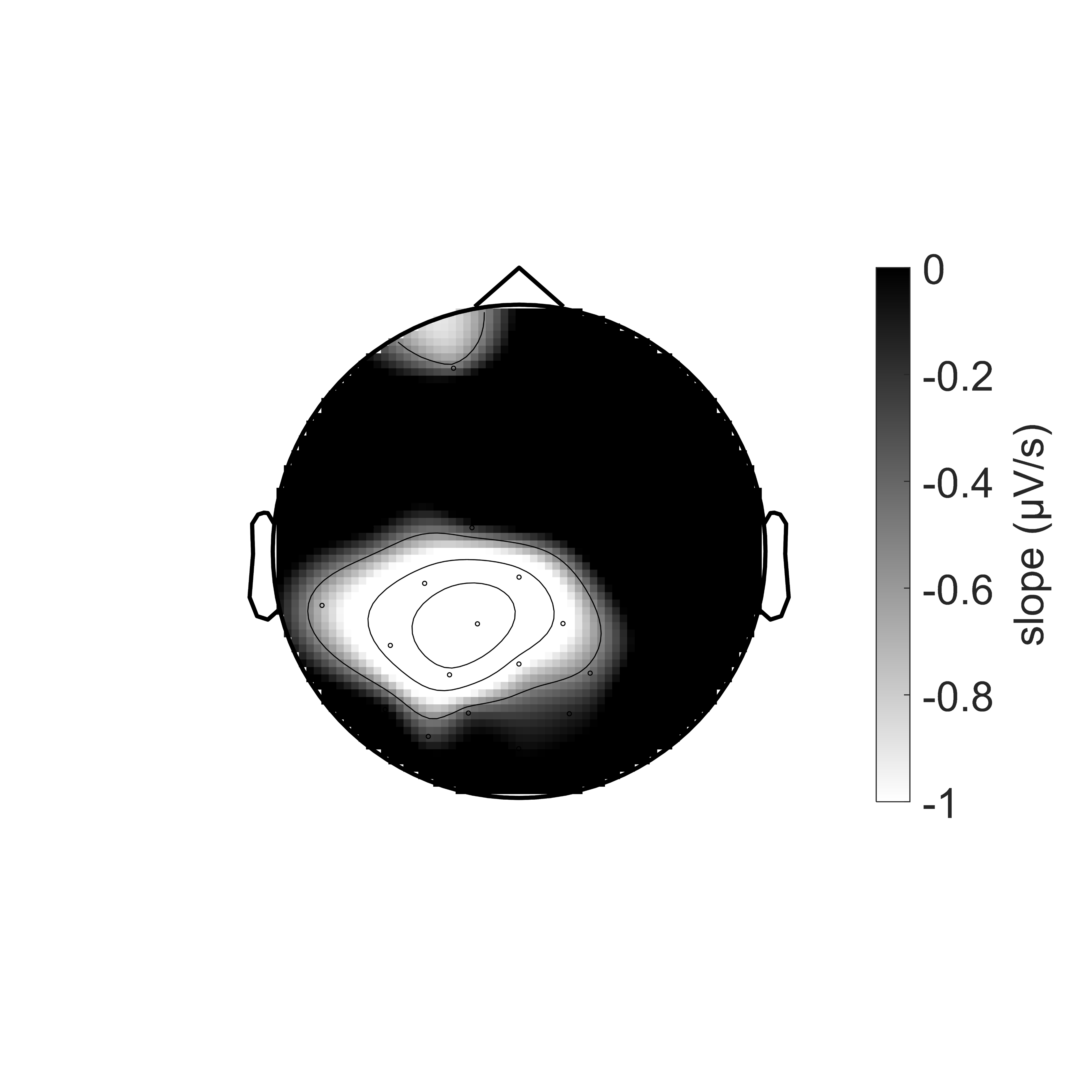

Supplement: Extended Data Figure 1-4 — Topography showing the slope of the ERPs in the motor-only condition, estimated with a linear regression analysis (see Materials and Methods) in the temporal window between –0.5 and –0.02 s. The topography of the effect is very similar to the visuomotor condition (compared to Fig. 1B). Download Figure 1-4, TIF file. [file enu-eN-NWR-0085-22-s05.tif]

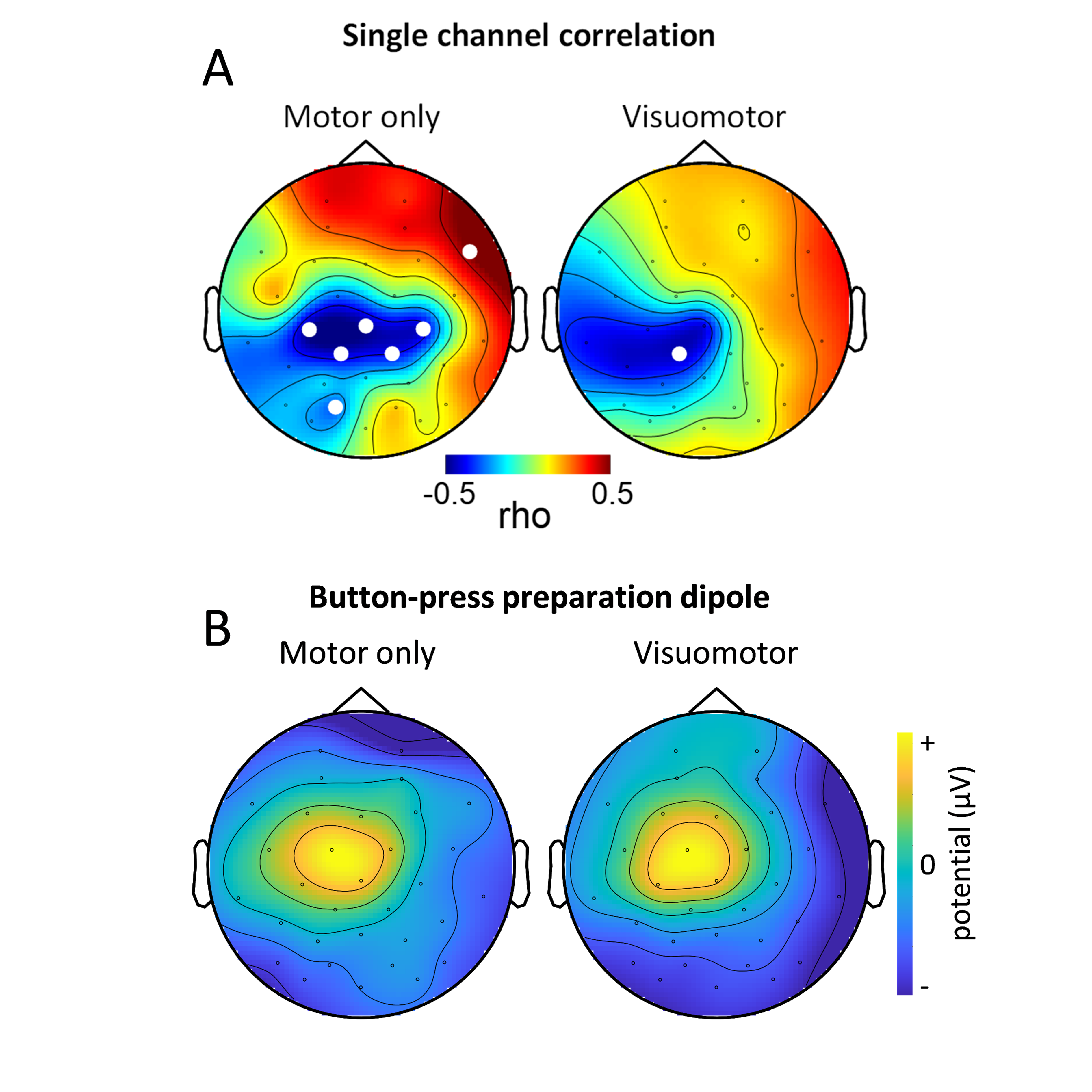

Supplement: Extended Data Figure 2-1 — Single channel correlation. A, Topographic map of the Pearson’s correlation coefficient between the readiness potential amplitude and the motor-induced modulation of visual accuracy, calculated for each single electrode; electrodes reaching significant correlations are marked in white (p > 0.05, uncorrected). For the motor-only condition (left) a cluster of centro-parietal electrodes (C3, CZ, CP1, CP2, PO3) negatively correlated with the magnitude of the behavioral modulation, while only one frontal electrode (F8) positively correlated with it. For the visuomotor condition (right), a centro-posterior electrode (CP1) negatively correlated with the magnitude of the perceptual modulation, no other channels reached statistical significance. B, Topographic map of the ERP grand-average scalp distribution in the time window between –0.5 and –0.1 s from button press, for the motor-only (left) and visuomotor tasks (right). The maps reveal the presence of an oriented dipole, with a negativity over front-temporal electrodes, right hemisphere. The grand-average ERPs computed at the electrodes FC1 (the focus of the positive activation over the left hemisphere) and F8 (the focus of the negative activation over the right hemisphere) are significantly anticorrelated (p < 0.001), suggesting the presence of an oriented dipole in the EEG signal driving the opposite correlation emerging in panel A. Download Figure 2-1, TIF file. [file enu-eN-NWR-0085-22-s06.tif]

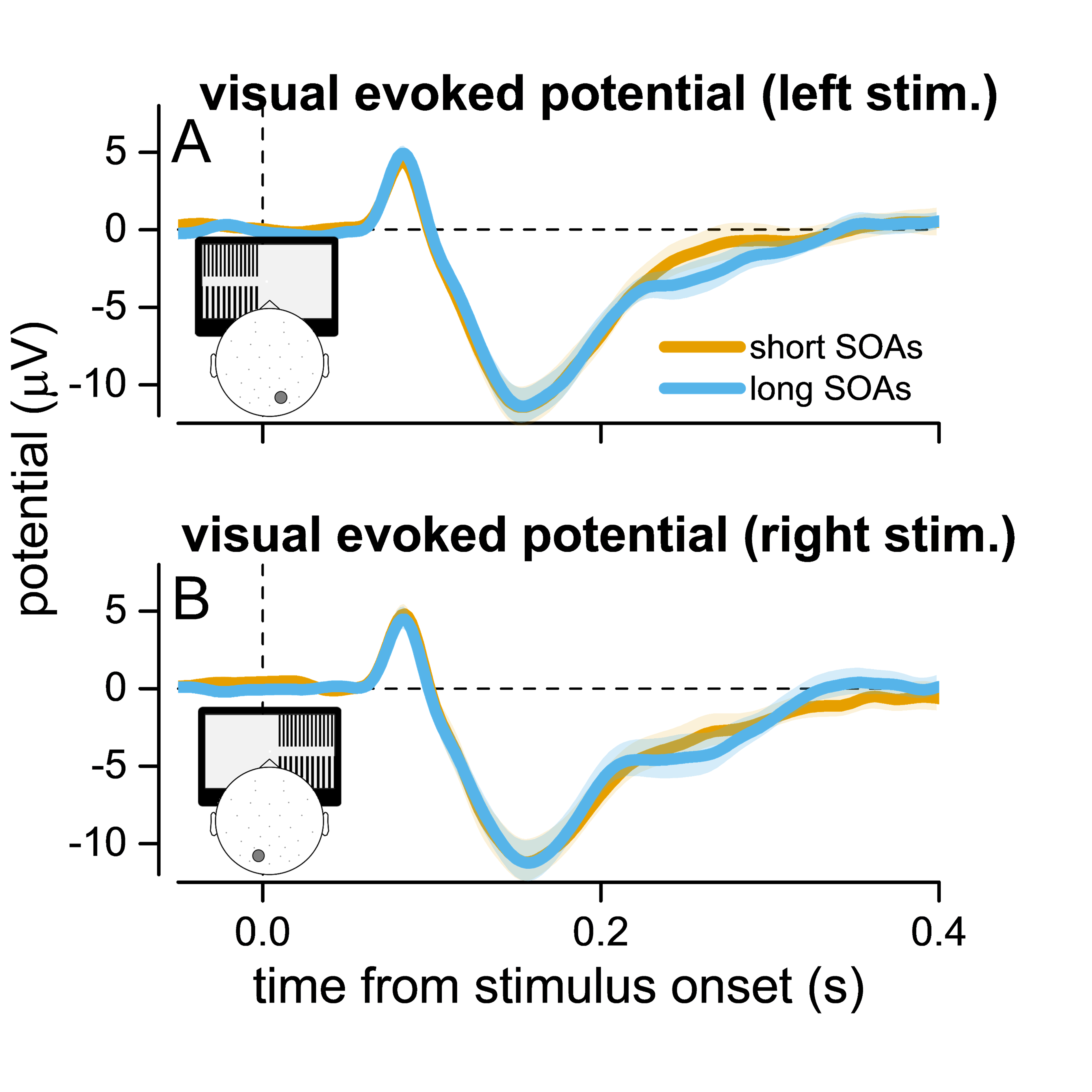

Supplement: Extended Data Figure 2-2 — Analyses on the visual-evoked potentials. A, Grand-average of ERPs for short (orange) and long ASIs trials (light blue) at the electrode PO4, for trials presented to the left visual field. B, Same as in A but for trials presented to the right visual field, at PO3. Colored shaded areas indicate the standard error. For each participant, the average mean voltage of the VEP was computed over the parietal-occipital electrode contralateral to the side of visual stimulation (i.e., PO4/PO3 for stimuli presented on the left/right visual field, respectively), after removing 100 ms of prestimulus baseline. Contralateral left and right responses were then pooled together. A two-tail paired t test, comparing short and long ASIs, was run for each datapoint, and p-values were FDR corrected (q = 0.05). A significant difference emerged between the two conditions at around 250–270 ms (uncorrected p = 0.04); however, it did not survive FDR correction (pFDR > 0.05). A closer look at the topology of the EEG activity in that temporal window revealed the presence of nonlateralized, central, positive component peaking at around 250 ms, and mostly expressed over CZ (data not shown). Despite the interesting and preliminary finding at 250 ms, the primary components associated with early visual responses are not modulated differently as a function of the ASI from action onset. Finally, we pulled together the two ASIs to compare the ERPs of correct versus incorrect trials. No difference was found between the two VEPs (p > 0.05, uncorrected; data not shown). Given that the VEP response is elicited by the simultaneous presentation of high-contrast gratings in both the upper and lower visual field stimuli, it is likely that the elicited responses are being saturated, making it difficult to measure a modulation between late short and long ASI stimuli. Several papers reported a reduction in certain visual evoked components (namely, N1 and P2; Schafer and Marcus, 1973; Gentsch and Schütz-Bosbach, 201 [file enu-eN-NWR-0085-22-s07.tif]
